# Treatment of hemolymphangioma of the spleen by laparoscopic partial splenectomy: a case report

**DOI:** 10.1186/1477-7819-12-60

**Published:** 2014-03-21

**Authors:** Yue Zhang, Xue-Min Chen, Dong-Lin Sun, Chun Yang

**Affiliations:** 1 Department of Hepatobiliary Surgery, The Third Affiliated Hospital of Soochow University, Changzhou 213003, China

## Abstract

Hemolymphangioma is a malformation of both lymphatic and blood vessels. The incidence of splenic hemolymphangioma is extremely rare. Laparoscopic partial splenectomy is feasible, reproducible, and safe in children with focal splenic tumors. We report on the case of a 12-year-old male with a large splenic hemolymphangioma successfully managed by laparoscopic partial splenectomy. The patient recovered well after operation. Laparoscopic partial splenectomy is a safe and minimally invasive technique for treatment of splenic hemolymphangioma located in the pole of the spleen.

## Background

Focal splenic tumors, including splenic cysts and vascular neoplasms, are uncommon and require complete resection to prevent complications or recurrence [1]. Hemolymphangioma is a malformation of both lymphatic and blood vessels. The incidence of splenic hemolymphangioma is extremely rare. In the past, total splenectomy has been the method of choice for the treatment of splenic tumors. Today, a spleen-preserving minimally invasive approach is being increasingly advocated, especially in children and young adults, in order to avoid overwhelming post-splenectomy infection [2,3]. With a better understanding of vascular anatomy and the development of fine laparoscopic skills, laparoscopic partial splenectomy became safe and feasible. In this paper, we report on a rare case of splenic hemolymphangioma successfully managed by laparoscopic partial splenectomy.

## Case presentation

**Figure 1:**
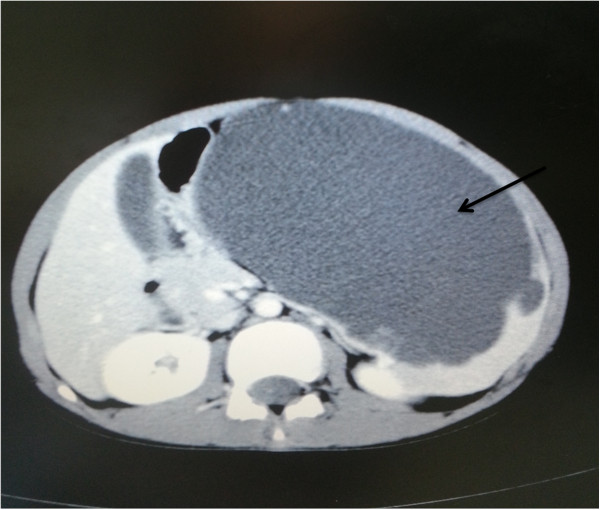
Computed tomography scans suggestive of a hypodense cystic lesion arising from the spleen.

**Figure 2:**
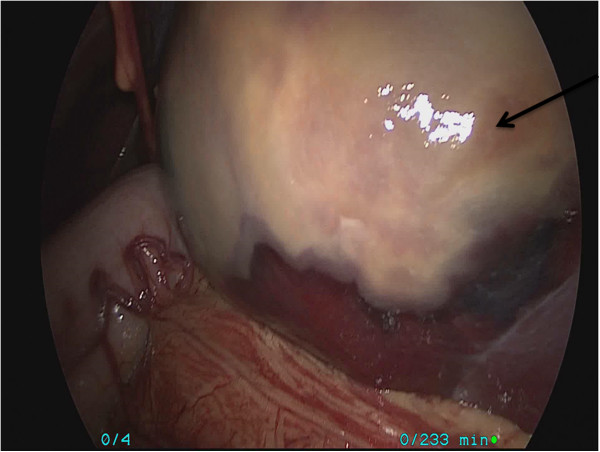
Splenic hemolymphangioma.

A 12-year-old male was admitted to our hospital with a complaint of abdominal pain for one month. There was no history of trauma, no weight loss, and no family history of cancer. A physical examination showed a tender left upper-quadrant abdominal mass. Laboratory tests, including serum tumor markers, were normal. Computed tomography confirmed a large 15.7 × 8.5 cm cyst occupying the upper pole of the spleen (Figure 1) and demonstrated almost total displacement of the remaining splenic parenchyma (Figure 2).

**Figure 3:**
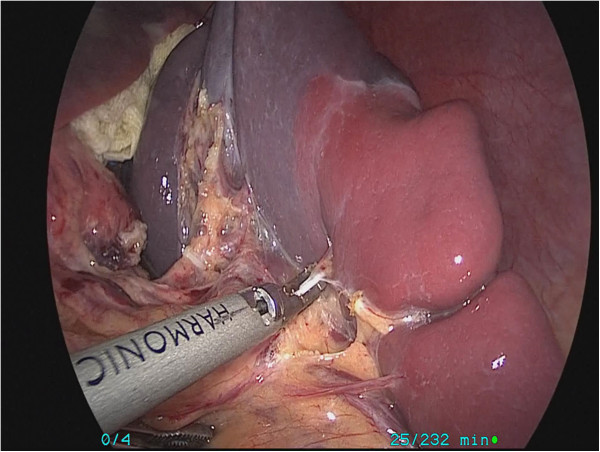
A well-defined line of demarcation at the upper pole after vessels were clipped.

**Figure 4:**
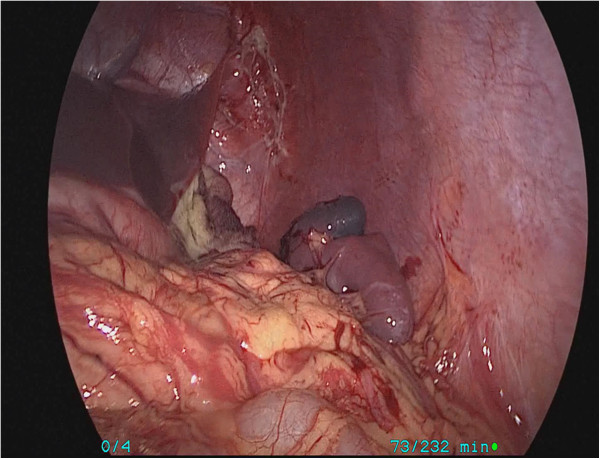
Complete excision of splenic hemolymphangioma.

The patient underwent a laparoscopic partial splenectomy. In brief, the patient was induced under general anesthesia and placed in the supine position. Using Hasson’s technique, the pneumoperitoneum was achieved at a pressure of 8 mmHg and a flow rate of 1.5 L/min. A 10-mm port was placed sub-umbilically for laparoscopy. Four more ports were placed under vision: a 5-mm port in the subcostal border in the right mid-clavicular line, a 5-mm port under the costal border 6 cm in the right mid-clavicular line, a 5-mm port parallel to the umbilicus in the left anterior axillary line, and a 5-mm port parallel to the umbilicus in the left mid-clavicular line. A hilar dissection was carefully performed, and the superior lobar vessels going toward the superior pole and cyst were identified and dissected. The vessels were clipped, which resulted in a well-defined line of demarcation at the upper pole (Figure 3). The cyst was punctured with the needle and about 800 mL of clear fluid was aspirated. After the cyst was evacuated and opened, using an UltraCision Harmonic Scalpel® (Ethicon Endo-Surgery Inc., USA), the upper pole of the spleen, along with the cyst, was resected keeping a 5-mm rim of devascularized splenic tissue (Figure 4). A suction drain was introduced through the anterior axillary line port and positioned lateral to the upper pole of the spleen. Intraoperative blood loss was 80 mL, and the operating time was 105 minutes.

**Figure 5:**
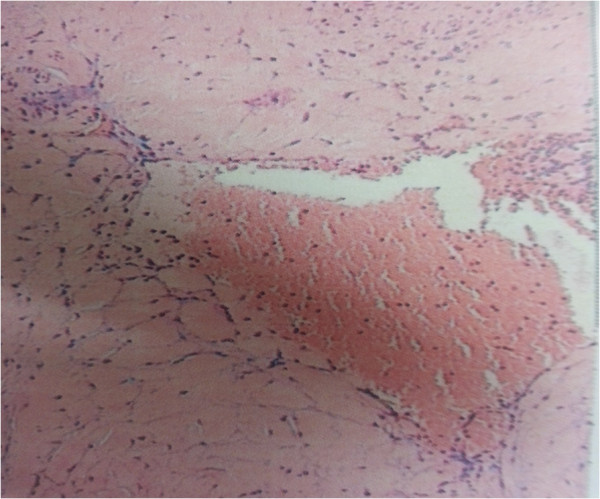
Pathological examination of the splenic hemolymphangioma (HE staining, 100×).

No post-splenectomy infectious complications occurred. His postoperative course was uneventful. The patient is asymptomatic on a follow-up of 1 year with no recurrence on ultrasonography and a normal platelet count. Microscopically, some dysplastic lymphatic vessels and blood vessels were observed in the tumor. The pathological diagnosis was hemolymphangioma (Figure 5).

## Discussion

Hemolymphangioma is a congenital malformation of the vascular system [4]. The formation of this tumor may be explained by obstruction of the venolymphatic communication between dysembryoplastic vascular tissue and systemic circulation; only one case has been reported previously [5]. Although rare, splenic hemolymphangioma should be considered in the differential diagnosis when a cystic tumor of the spleen is found, particularly when there is insufficient evidence to diagnose cystic hemangiomas, cystic lymphangiomas, and epidermoid and dermoid cysts. The optimal treatment strategy remains controversial. The surgical options are based on the size of the tumor, its relation to the splenic hilar vessels and parenchyma, and the amount of healthy splenic tissue remaining. The aim of treatment is to remove the entire tumor, sparing splenic tissue and avoiding recurrence. In a multicenter study performed in four European countries [6], a high recurrence rate was associated with laparoscopic fenestration and deroofing, and laparoscopic total cyst removal with or without splenic tissue has been considered as the treatment of choice. Additionally, deroofing carries the risk of content spillage. Currently, laparoscopic partial splenectomy is feasible, reproducible, and safe in children with hematological diseases or focal splenic tumors. Partial splenectomy is the best method to prevent post-splenectomy infections since it preserves the spleen’s immune role. Laparoscopic partial splenectomy also offers the benefits of a minimally invasive approach: laparoscopy is now considered the gold standard for total splenectomy in children [7-9]. In fact, laparoscopy leads to fewer complications such as wound dehiscence, infections, intussusceptions, and pleural effusions. Less postoperative adhesion facilitates a second laparoscopy if completions of the splenectomy or cholecystectomy are required. According to Minkes [10], laparoscopic splenectomy in children can be performed safely with a low conversion rate (2.9%). Laparoscopic partial splenectomy is still a challenging procedure (e.g., bleeding from the cut edge of the spleen can be difficult to control). Nevertheless, with an understanding of the vascular anatomy of the spleen, it can be safely performed. Based on vascular distribution, the spleen is divided mainly into two lobes. In our case, when the upper lobe vessels were ligated, the line of demarcation was formed making it easily accessible for partial splenectomy without major blood loss.

## Conclusions

In conclusion, laparoscopic partial splenectomy is a safe and minimally invasive technique for the treatment of splenic hemolymphangioma located at the pole of the spleen.

## Consent

Written informed consent was obtained from the patient’s parents for the publication of this report and any accompanying images.

## Competing interests

The authors declare that they have no competing interests.

## Authors’ contributions

YZ and X-MC drafted and revised the manuscript. D-LS and Y C were responsible for acquisition and interpretation of data. All authors read and approved the final manuscript.
